# Peer perceptions of clinicians using generative AI in medical decision-making

**DOI:** 10.1038/s41746-025-01901-x

**Published:** 2025-08-18

**Authors:** Haiyang Yang, Tinglong Dai, Nestoras Mathioudakis, Amy M. Knight, Yuna Nakayasu, Risa M. Wolf

**Affiliations:** 1 Carey Business School, Johns Hopkins University, Baltimore, MD USA; 2 Hopkins Business of Health Initiative, Johns Hopkins University, Washington, DC USA; 3 School of Nursing, Johns Hopkins University, Baltimore, MD USA; 4 Data Science and AI Institute, Johns Hopkins University, Baltimore, MD, USA; 5 School of Medicine, Johns Hopkins University, Baltimore, MD USA; 6 Bloomberg School of Public Health, Johns Hopkins University, Baltimore, MD USA

**Keywords:** Business, Human behaviour, Health care economics

## Abstract

This study investigates how a physician’s use of generative AI (GenAI) in medical decision‑making is perceived by peer clinicians. In a randomized experiment, 276 practicing clinicians evaluated one of three vignettes depicting a physician: (1) using no GenAI (Control), (2) using GenAI as a primary decision-making tool (GenAI-primary), and (3) using GenAI as a verification tool (GenAI-verify). Participants rated the physician depicted in the GenAI‑primary condition significantly lower in clinical skill (on a 1–7 scale; mean = 3.79) than in the Control condition (5.93, *p* < 0.001). Framing GenAI use as verification partially mitigated this effect (4.99, *p* < 0.001). Similar patterns appeared for perceived overall healthcare experience and competence. Participants also acknowledged GenAI’s value in improving accuracy (4.30, *p* < 0.002) and rated institutionally customized GenAI more favorably (4.96, *p* < 0.001). These findings suggest that while clinicians see GenAI as helpful, its use can negatively impact peer evaluations. These effects can be reduced, but not fully eliminated, by framing it as a verification aid.

## Introduction

The emergence of generative artificial intelligence (GenAI) systems has generated increasing interest in their potential to enhance healthcare delivery since the introduction of ChatGPT in November 2022^1^. As of early 2024, more than 70% of healthcare organizations are either pursuing or have already incorporated GenAI into their healthcare workflows^2^. GenAI offers significant promise in supporting physicians by streamlining clinical decision-making through the rapid analysis of patient data. While much attention has focused on using GenAI to enhance efficiency and reduce burdens associated with electronic medical records^3–5^, studies have also explored its role in *medical decision-making*, from generating differential diagnoses with clinical vignettes^6^ to improving decision-support tools integrated into electronic medical record systems^7,8^.

Efforts to incorporate computerized tools, including medical AI, into medical decision-making date back several decades. Early examples, such as MYCIN in the 1970s, used rule-based expert systems to recommend treatments for infectious diseases but faced challenges in usability and clinical uptake^9^. IBM’s Watson represented a more recent attempt to augment medical decision-making through machine learning applications, particularly in oncology, but encountered mixed results due to data and implementation complexities and inconsistent clinical impact^10^. In contrast, GenAI marks a major shift, with its ability to process freeform, unstructured data, produce human-like responses, and provide rapid insights, offering a more flexible and accessible tool for decision support.

Despite its potential, real-world applications of GenAI in medical decision-making remain limited^11^. One potential barrier to broader adoption is the impact of physicians’ reputational concerns among their peers^12^. Prior studies, often in the form of laboratory experiments among trainees or non-medical practitioners, show physicians hold less favorable views of their peers who use computerized tools in patient care^13–15^. However, little is known about how *practicing* clinicians perceive peers who use decision-support tools, including GenAI, for medical decision-making. Understanding these perceptions is important because peer reputation influences professional success: In healthcare, patients frequently lack the ability to evaluate physician quality independently^16^, and they often rely on referrals from trusted intermediaries, such as primary care physicians, triage nurses, and specialists, to select experts^17^. Peer reputation not only shapes these referral networks but also plays a key role in the adoption of medical technologies^18^.

This study examines how a physician’s use of GenAI in medical decision-making is perceived by peer clinicians, focusing on key dimensions such as competence, clinical skills, and overall healthcare experience. Using a controlled survey experiment, we evaluated these perceptions across different scenarios, including GenAI as a primary decision-making tool and GenAI framed as a verification tool. By linking these perceptions to broader issues of professional reputation and referral patterns, this study highlights the nuanced interaction between technology adoption and peer evaluations. It also sheds new light on the integration of GenAI into clinical workflows and the challenges of balancing innovation with professional trust.

Prior research suggests that seeking advice or assistance can lead to perceived competence penalties, even when the advice enhances decision quality; these effects appear to be attenuated when the advice-seeker signals humility or deference to social norms^19^. In clinical contexts, physicians’ use of GenAI may similarly be interpreted as a signal of lower personal competence. This possibility aligns with a broader body of advice-taking literature, which shows that reliance on external input can be perceived as a weakness rather than a strength^20^.

Based on these insights, we formulated two key hypotheses. First, we hypothesized that, compared with those who do not use GenAI, physicians who use GenAI as a primary decision-making tool would be perceived as having lower clinical skills, providing a worse overall healthcare experience, and being less competent overall. Second, we hypothesized that presenting GenAI as a verification tool, rather than as a primary decision-making tool, would partially—but not fully—mitigate these negative perceptions.

## Results

A total of 276 clinicians participated in the study, including 178 physicians, 28 fellows/residents, 60 advanced practice providers (physician assistants and nurse practitioners), and 10 individuals in other clinical roles. An additional 123 individuals started the survey but did not complete it and thus were not included in the analysis. In the total cohort, most participants were aged 35–54 years; 60.1% were female, 19.2% Asian, 4.7% Black, and 62.3% White. As shown in Table 1, participants were balanced across years of practice experience and practice setting (inpatient and outpatient). Baseline demographic and workforce characteristics did not differ significantly across the three conditions. For clarity, the “GenAI-primary” condition refers to a physician using GenAI as the primary decision-making aid, whereas in the “GenAI-verify” condition, the physician uses GenAI only to verify their decision. A summary of participants’ responses is provided in Table 2.

**Table 1:** Characteristics of study participants by experimental condition

Factor	Level	Control	GenAI-primary	GenAI-verify	*p*-value
N		90	93	93	
Age	Prefer not to answer	2 (2%)	3 (3%)	2 (2%)	0.68
	25–34	11 (12%)	17 (18%)	17 (18%)	
	35–44	27 (30%)	33 (35%)	28 (30%)	
	45–54	25 (28%)	20 (22%)	26 (28%)	
	55–65	16 (18%)	12 (13%)	17 (18%)	
	65 and over	9 (10%)	8 (9%)	3 (3%)	
Gender	Prefer not to answer	2 (2%)	4 (4%)	1 (1%)	0.27
	Female	59 (66%)	57 (61%)	50 (54%)	
	Male	29 (32%)	31 (33%)	42 (45%)	
	Non-binary	0 (0%)	1 (1%)	0 (0%)	
Race	Prefer not to answer	3 (3%)	8 (9%)	8 (9%)	0.92
	Asian	15 (17%)	21 (23%)	17 (19%)	
	Black or African American	4 (5%)	4 (4%)	5 (5%)	
	White	59 (69%)	55 (59%)	58 (64%)	
	Other	1 (1%)	1 (1%)	1 (1%)	
	More than one race	4 (5%)	4 (4%)	2 (2%)	
Ethnicity	Non-Hispanic	86 (96%)	93 (100%)	90 (97%)	0.14
	Hispanic or Latino	4 (4%)	0 (0%)	3 (3%)	
Number of patients/week	10 or less	17 (19%)	16 (17%)	12 (13%)	0.61
	11–20	21 (23%)	18 (19%)	28 (30%)	
	21–30	22 (24%)	23 (25%)	19 (20%)	
	31–40	10 (11%)	13 (14%)	12 (13%)	
	41–50	5 (6%)	8 (9%)	12 (13%)	
	50 or more	15 (17%)	15 (16%)	10 (11%)	
Years in practice	Prefer not to answer	1 (1%)	3 (3%)	1 (1%)	0.65
	5 years or less	20 (22%)	24 (26%)	26 (28%)	
	6–15 years	24 (27%)	33 (35%)	30 (32%)	
	16–25 years	26 (29%)	18 (19%)	20 (22%)	
	Over 25 years	19 (21%)	15 (16%)	16 (17%)	
Clinical Service	Inpatients	26 (29%)	27 (29%)	30 (32%)	0.37
	Outpatients	32 (36%)	42 (45%)	30 (32%)	
	Both Equally	32 (36%)	24 (26%)	33 (35%)	
Clinician type	Physician	66 (73%)	70 (75%)	70 (75%)	0.75
	Advanced Practice Provider	22 (24%)	18 (19%)	20 (22%)	
	Other	2 (2%)	5 (5%)	3 (3%)

**Table 2:** Summary of participants’ ratings by experimental condition

	Clinical Skills	Overall Healthcare Experience	Overall Competence	GenAI Usefulness	Custom GenAI Usefulness
Control	5.93 (1.24)	4.48 (0.82)	5.99 (1.25)	4.47 (1.57)	4.83 (1.62)
GenAI-primary	3.79 (1.62)	3.08 (1.30)	3.71 (1.61)	4.10 (1.55)	4.84 (1.58)
GenAI-verify	4.99 (1.67)	3.72 (1.24)	4.94 (1.74)	4.35 (1.83)	5.19 (1.73)

Mean ratings (with standard deviations in parentheses) for Clinical Skills, Overall Healthcare Experience, Overall Competence, GenAI Usefulness, and Custom GenAI Usefulness across three conditions (Control, GenAI-primary, and GenAI-verify). Ratings were provided using 7-point Likert-style scales, except for Overall Healthcare Experience, which used a 5-point “star” rating scale.

### Clinical Skills

**Fig. 1:**
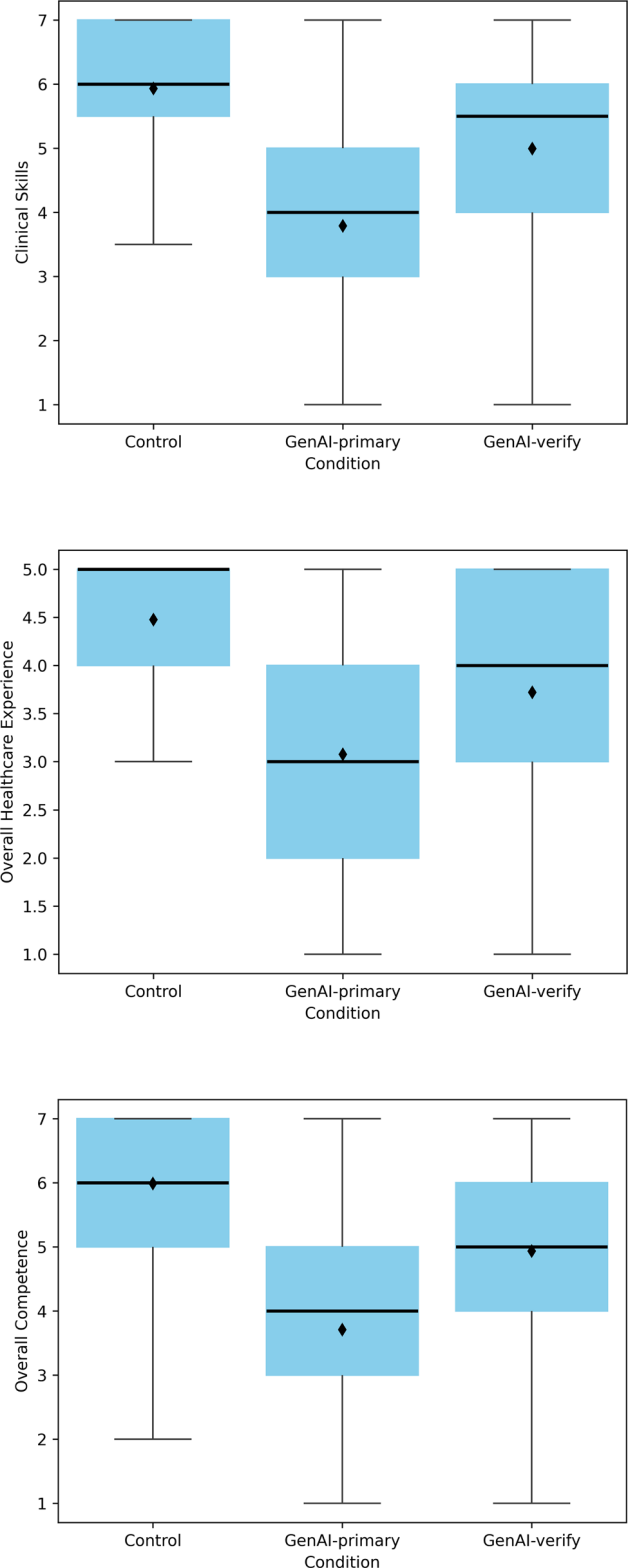
Clinicians’ evaluations of clinical skill, overall healthcare experience, and overall competence across conditions. Clinicians (*n* = 276) rated the physician of their assigned condition: Control (no GenAI), GenAI-primary (using GenAI as a primary decision-making tool), or GenAI-verify (using GenAI as a verification tool). Boxes represent the interquartile range (IQR), with horizontal lines indicating medians and diamonds indicating means. Whiskers extend to responses within 1.5 IQRs of the lower and upper quartiles.

Ratings of clinical skills differed significantly across the three conditions (F(2, 273) = 45.45, *p* < 0.001, η_p_² = 0.25; Fig. 1, first panel**)**. The mean (SD) clinical skills score for the Control condition was 5.93 (1.24), for GenAI-primary was 3.79 (1.62), and for GenAI-verify was 4.99 (1.67). The difference between the GenAI-primary and Control conditions was statistically significant (F(1, 273) = 90.30, *p* < 0.001, η_p_² = 0.25), as was the difference between GenAI-verify and Control conditions (F(1, 273) = 17.33, *p* < 0.001, η_p_² = 0.06). Presenting GenAI as a verification tool partially mitigated this effect, though the clinical skills rating remained lower than in the Control condition (F(1, 273) = 28.99, *p* < 0.001, η_p_² = 0.10).

### Overall Healthcare Experience

Evaluations of overall healthcare experience differed significantly across the three conditions (F(2, 273) = 34.38, *p* < 0.001, η_p_² = 0.20; Fig. 1, second panel). The mean (SD) evaluations were 4.48 (0.82) in the Control condition, 3.08 (1.30) in the GenAI-primary condition, and 3.72 (1.24) in the GenAI-verify condition. Compared with those in the Control condition, evaluations in the GenAI-primary condition (F(1, 273) = 68.67, *p* < 0.001, η_p_² = 0.20) and GenAI-verify condition (F(1, 273) = 20.02, *p* < 0.001, η_p_² = 0.07) were significantly lower. The healthcare experience was rated significantly lower in the GenAI-primary condition than in the GenAI-verify condition (F(1, 273) = 14.77, *p* < 0.001, η_p_² = 0.05). That is, while presenting GenAI as a verification tool improved healthcare experience evaluations, they remained lower than those in the Control condition.

Mediation analysis revealed that clinical skills ratings mediated the relationship between study conditions and healthcare experience evaluations. This analysis showed the relative indirect effect of D_GenAI-primary_ through clinical skill ratings was significant (β = −1.30, SE = 0.15, 95% CI: [−1.59, −1.01]), and the relative indirect effect of D_GenAI-verify_ was also significant (β = −0.57, SE = 0.13, 95% CI: [−0.83, −0.31]). In other words, generative AI usage reduced the ratings of the physician’s clinical skills, which in turn negatively impacted the evaluations of the overall healthcare experience provided by the physician.

### Overall Competence

Overall competence evaluations differed significantly across the three conditions (F(2, 273) = 49.60, *p* < 0.001, η_p_² = 0.27; Fig. 1, third panel). The mean (SD) ratings were 5.99 (1.25) in the Control condition, 3.71 (1.61) in the GenAI-primary condition, and 4.94 (1.74) in the GenAI-verify condition. Compared with those in the Control condition, competence evaluations were significantly lower in the GenAI-primary condition (F(1, 273) = 98.91, *p* < 0.001, η_p_² = 0.27) and GenAI-verify condition (F(1, 273) = 21.13, *p* < 0.001, η_p_² = 0.07) conditions. Competence evaluations in the GenAI-primary condition were significantly lower than those in the GenAI-verify condition (F(1, 273) = 29.09, *p* < 0.001, η_p_² = 0.10). That is, presenting GenAI as a verification tool improved competence evaluations, but they remained significantly lower than in the Control condition.

Mediation analysis revealed that clinical skills ratings mediated the relationship between study conditions and competence evaluations. The relative indirect effect of D_GenAI-primary_ through clinical skill ratings was significant (β = −1.93, SE = 0.20, 95% CI: [−2.33, −1.55]), and the relative indirect effect of D_GenAI-verify_ was also significant (β = −0.85, SE = 0.20, 95% CI: [−1.24, −0.46]). The use of GenAI decreased ratings of the physician’s clinical skills, which in turn led to lower competence evaluations.

### Perceived Usefulness of GenAI

The perceived usefulness of GenAI technologies did not differ across the three conditions. Participants rated GenAI technologies as useful for ensuring clinical assessment accuracy (mean [SD], 4.30 [1.65]; *t* = 3.06, *p* < 0.002, Cohen’s d = 0.18), and they rated customized GenAI as even more useful (mean [SD], 4.96 [1.65]; *t* = 9.64, *p* < 0.001, Cohen’s d = 0.58). That is, participants perceived GenAI as a useful tool for clinical assessment.

## Discussion

In a study of 276 clinicians at a major hospital system, we found that while clinicians acknowledge the potential of GenAI to enhance medical decision-making, they consistently rate physicians using such tools as being less clinically skilled, less competent, and delivering a lower quality healthcare experience. Although framing GenAI usage as a verification tool reduces some of these negative perceptions, it does not fully mitigate them. These findings carry significant implications for the development and deployment of AI tools in medicine, particularly as they relate to physician perceptions and patient care experience.

Our findings align with advice-taking theory^19,20^, suggesting that reliance on external input, such as GenAI, can trigger penalties on perceived competence. Observers may apply attributional discounting, attributing the physician’s success more to the AI tool and less to the physician’s actual abilities^21^. From a reputation signaling perspective, visible utilization of GenAI may undermine a physician’s perceived clinical expertise among peers^22^. These dynamics may explain why even framing GenAI use as verification did not fully restore peer evaluations to baseline levels.

To our knowledge, this is the first study to examine clinicians’ perceptions of medical decision-making and healthcare experience in the context of GenAI. A strength of this study was the use of a between-participants design with three clinical scenarios to evaluate whether the use of GenAI for medical decision-making influences clinicians’ perceptions of care quality. It has been said that clinicians who use AI will replace those who do not use AI^22^; it is thus important to understand the implications of clinical AI utilization. General use of medical decision-support tools augments the expertise of clinicians, and can lead to greater diagnostic accuracy, potentially improving the capabilities of clinicians and enhancing their ability to provide evidence-based care^23^. Historically, the perception of clinical expertise was characterized by competence in clinical skills, advanced clinical judgment, cognitive abilities, a deep understanding of clinical reasoning and diagnostic processes, and scholarship^24,25^. This is aligned with our findings that clinicians perceived the physician who did not use GenAI to have superior clinical skills, whereas physicians who relied on GenAI for medical decision-making were perceived as less competent in their clinical skills. Given the increasing use of AI in medicine, this perception is likely to change in the coming years, but not without challenges along the way.

While there are benefits to computerized and GenAI-based decision support systems, successful implementation of these tools will require overcoming clinician and institutional resistance, and modifying perceptions of clinical expertise with use of these systems. The practicing clinician still needs to leverage their clinical acumen within the context of the specific clinical situation, but the decision-making process can be further refined and supported through the integration of GenAI clinician-decision support tools that enhance precision and efficiency. As GenAI reshapes medical decision support, clinicians must remain open to adopting innovative systems that are rigorously validated for efficacy and safety, ensuring they complement clinical expertise while improving patient care outcomes.

In addition, using GenAI as the initial decision-maker can introduce confirmation bias, a cognitive tendency to favor information that confirms one’s initial hypothesis^26,27^. In practice, relying on GenAI first may make it cognitively harder for clinicians to consider alternative diagnoses or treatments, potentially leading to overreliance. By contrast, using GenAI as a verification tool—after formulating one’s own plan—may help mitigate this bias. This important consideration further differentiates the two GenAI use cases.

This study has several limitations. First, while the respondents included a variety of clinicians, the majority were physicians, and thus results may not be generalizable to all clinicians within a hospital system. Second, the diabetic care scenarios used in the study were developed and prescreened by clinicians to ensure both realism and readability. The amount of time participants spent responding to their assigned healthcare scenario did not differ across the three conditions (*p* > 0.30), indicating that the effects and mediational patterns observed in the study could not simply be attributed to differences in effort or comprehension. Future research utilizing different scenarios and different measures can further examine the generalizability of our findings. Third, given the population we examined (i.e., frontline clinicians), we did not have an opportunity to perform a separate formal instrument development or validation for our survey constructs prior to data collection. Our measures (e.g., clinical skills, healthcare experience) were chosen based on literature and expert input, and we treated the Likert-scale responses as approximately continuous for analysis, an approach not uncommon in survey experiments^28,29^, but these choices may introduce measurement limitations. Future studies should employ rigorous scale development and validation to refine the measurement approach. Fourth, this study utilized a convenience sample, yet the research still offers novel data on the perspective of clinicians on use of GenAI for medical decision-making. Fifth, this study was conducted at one health system, and findings at other health systems in other parts of the country or the world may be different. Finally, our study focused exclusively on peer perceptions among clinicians. Future studies should examine how patients perceive clinicians’ use of GenAI technologies, and how these perceptions influence trust, adherence, and clinical outcomes. Patients’ trust and acceptance of clinicians’ recommendations may be influenced differently by GenAI usage^30^, with important implications for adherence and outcomes.

In conclusion, this study suggests that although clinicians clearly perceive GenAI technologies as beneficial for optimal medical decision-making, they evaluate their peers who utilize such technologies as lower in clinical skills, less effective in providing an optimal healthcare experience, and less competent. Presenting GenAI technologies as tools for verification purposes can help reduce these negative evaluations, but does not eliminate them. Although these findings highlight that there may be challenges with the adoption and increasing use of GenAI in medicine, they also emphasize the importance of thoughtful approaches to its implementation in healthcare settings.

## Methods

Johns Hopkins Medicine is a six-entity health system with four hospitals in Maryland, one in Washington, D.C., and one in Florida. Clinicians at these hospitals include attending physicians, residents, fellows, and advanced practice providers (APPs), such as physician assistants and nurse practitioners. The survey invitation was distributed via a departmental listserv that included approximately 4000 clinicians across various specialties, encompassing physicians, APPs, and other clinical staff. Clinicians at the two academic hospitals in Baltimore, MD, were invited to participate in the study, which was conducted using the Qualtrics survey tool. We conservatively assumed that the effect sizes would be relatively moderate. Using G*Power Version 3.1.9.7 (*f* = 0.2, power = 0.8, *α* = 0.05, omnibus one-way ANOVA with 3 conditions), we estimated the minimum sample size per condition to be about 82 participants per condition (i.e., a minimum sample size of 246 participants in total). The survey link was sent weekly for three consecutive weeks in August 2024. To ensure privacy, IP and other personally identifiable information were not tracked in the study. The survey was open between August 1, 2024, and September 10, 2024, with participation being voluntary and based on availability and willingness, thereby forming a convenience sample. There were no restrictions on inclusion based on specialty or department, and no incentives were offered for survey completion. The study protocol was approved by the Johns Hopkins School of Medicine Institutional Review Board (JHM IRB00393208). Participants provided informed consent by proceeding past the introductory survey page, which described the study’s purpose, procedures, and their rights as participants, per the IRB-approved protocol.

Participants were randomly assigned to one of three conditions: **Control** (no GenAI involved), **GenAI-primary** (physician using GenAI for medical decision-making), or **GenAI-verify** (physician using GenAI to verify medical decision-making). In the GenAI-primary and GenAI-verify conditions, GenAI was explicitly referenced. In the Control condition, participants were presented with a clinical scenario in which a physician assesses a patient with diabetes and recommends a new antihyperglycemic medication *without* any mention or use of GenAI. In the GenAI-primary condition, the same scenario and recommendation were presented, but the physician was noted to have used GenAI during the decision-making process. In the GenAI-verify condition, the scenario and recommendation were identical to the GenAI‑primary scenario, but the use of GenAI was framed as “an additional level of verification.” The clinical scenarios (see Supplementary Note 1) were developed collaboratively with practicing physicians and pretested to ensure realism, clarity, and relevance to current clinical practice. The scenarios were iteratively refined with feedback from study collaborators and then independently reviewed by an internist, a medical student, and a surgeon, all of whom confirmed that the vignettes were clear and clinically realistic. The case involved adult diabetes management, a common and routine decision-making context selected to enhance ecological validity.

All participants completed the same set of measures. First, they rated the physician’s clinical skills using two Likert scale items: clinical management skills (1 = poor, 7 = excellent) and the appropriateness of the recommendation to start a new medication (1 = poor, 7 = excellent). This type of Likert scale approach has been used to evaluate physician performance and satisfaction in both clinical and training contexts^31^. Participants then provided two overall evaluations: the quality of the healthcare experience delivered by the physician (rated from 1 to 5 stars) and the physician’s competence as a medical doctor (1 = not competent at all, 7 = highly competent). The 5-star scale approach is not only commonly used in healthcare evaluations and satisfaction research^32,33^, but also widely used by hospitals and payers for public quality reporting and patient-experience benchmarking^34,35^. Participants also rated their perceptions of GenAI technologies on two items: the extent to which GenAI could help ensure the accuracy of the physician’s clinical assessment (1 = not at all, 7 = very much) and the extent to which GenAI customized for Johns Hopkins could enhance assessment accuracy (1 = not at all, 7 = very much). In addition, participants provided basic demographic information, including age and gender. Responses to the two clinical skill items were averaged to create a composite measure of clinical skills (*r* = 0.86, *p* < 0.001) for subsequent analyses.

### Statistical analysis

Summary statistics were calculated to describe participants’ demographic characteristics. We used analysis of variance (ANOVA) to compare responses across all three experimental conditions (Control, GenAI-primary, GenAI-verify), treating the 7-point Likert responses as approximately continuous measures for these analyses (as in similar survey experiments^28,29^), with post-hoc contrast analyses to assess pairwise differences. Tukey HSD and Bonferroni tests were conducted, which confirmed that all significant contrast results remained robust after corrections for multiple comparisons. Two-tailed t-tests were employed to assess deviations from indifference points.

To examine whether clinical skill ratings mediated the relationship between study conditions and the core dependent measures (healthcare experience evaluations and competence evaluations), two dummy variables were created: one for the GenAI-primary condition (D_GenAI-primary_) and one for the GenAI-verify condition (D_GenAI-verify_). A value of zero on both dummy variables represented the Control condition, which served as the reference group for comparisons. Two multicategorical mediation analyses were conducted (PROCESS Model 4; 5000 bootstrap resamples^36^) with D_GenAI-primary_ and D_GenAI-verify_ as the independent variables. SPSS Version 28 was used in these analyses. The figure was created using Python 3.11.

## Supplementary information

Supplementary Information

## Data Availability

The datasets generated and analyzed during this study are available from the corresponding author upon reasonable request.

## Code availability

No custom code was used in this study.

## References

[1] Minssen, T., Vayena, E. & Cohen, I. G. The challenges for regulating medical use of ChatGPT and other large language models. *JAMA* **330**, 315 (2023). 37410482 10.1001/jama.2023.9651

[2] Lamb, J., Israelstam, G., Agarwal, R. & Bhasker, S. *The Future of Generative AI in Healthcare*. https://www.mckinsey.com/industries/healthcare/our-insights/generative-ai-in-healthcare-adoption-trends-and-whats-next (2024).

[3] Ayers, J. W. et al. Comparing physician and artificial intelligence chatbot responses to patient questions posted to a public social media forum. *JAMA Intern Med* **183**, 589 (2023). 37115527 10.1001/jamainternmed.2023.1838 PMC10148230

[4] Tierney, A. A. et al. Ambient artificial intelligence scribes to alleviate the burden of clinical documentation. *NEJM Catalyst* **5** (2024).

[5] Tai-Seale, M. et al. AI-generated draft replies integrated into health records and physicians’ electronic communication. *JAMA Netw. Open* **7**, e246565 (2024). 38619840 10.1001/jamanetworkopen.2024.6565 PMC11019394

[6] Goh, E. et al. Large language model influence on diagnostic reasoning: a randomized clinical trial. *JAMA Netw. Open* **7**, e2440969 (2024). 39466245 10.1001/jamanetworkopen.2024.40969 PMC11519755

[7] Liu, S. et al. Using AI-generated suggestions from ChatGPT to optimize clinical decision support. *J. Am. Med. Inform. Assoc.* **30**, 1237–1245 (2023). 37087108 10.1093/jamia/ocad072 PMC10280357

[8] Rodriguez, D. V. et al. Leveraging generative AI tools to support the development of digital solutions in health care research: case study. *JMIR Hum. Factors* **11**, e52885 (2024). 38446539 10.2196/52885 PMC10955400

[9] Dai, T. & Abràmoff, M. D. Incorporating Artificial Intelligence into Healthcare Workflows: Models and Insights. in *Tutorials in Operations Research: Advancing the Frontiers of OR/MS: From Methodologies to Applications* 133–155. 10.1287/educ.2023.0257 (INFORMS, 2023).

[10] Strickland, E. IBM Watson, heal thyself: How IBM overpromised and underdelivered on AI health care. *IEEE Spectr.* **56**, 24–31 (2019).

[11] Yim, D., Khuntia, J., Parameswaran, V. & Meyers, A. Preliminary evidence of the use of generative AI in health care clinical services: systematic narrative review. *JMIR Med Inf.* **12**, e52073 (2024). 10.2196/52073 PMC10993141 38506918

[12] Dai, T. & Tayur, S. Designing AI-augmented healthcare delivery systems for physician buy-in and patient acceptance. *Prod. Oper. Manag.* **31**, 4443–4451 (2022).

[13] Arkes, H. R., Shaffer, V. A. & Medow, M. A. Patients derogate physicians who use a computer-assisted diagnostic aid. *Med Decis. Mak.* **27**, 189–202 (2007). 10.1177/0272989X06297391 17409368

[14] Wolf, J. R. Do IT students prefer doctors who use IT?. *Comput. Hum. Behav.* **35**, 287–294 (2014).

[15] Yin, Y., Jia, N. & Wakslak, C. J. AI can help people feel heard, but an AI label diminishes this impact. *Proc. Natl Acad. Sci. USA.* **121**, e2319112121 (2024). 38551835 10.1073/pnas.2319112121 PMC10998586

[16] Makary, M. *Unaccountable: What Hospitals Won’t Tell You and How Transparency Can Revolutionize Health Care*. (Bloomsbury Publishing USA, New York, 2013).

[17] Harris, K. M. How do patients choose physicians? Evidence from a national survey of enrollees in employment-related health plans. *Health Serv. Res.* **38**, 711–732 (2003). 12785569 10.1111/1475-6773.00141 PMC1360911

[18] Navathe, A. & David, G. The formation of peer reputation among physicians and its effect on technology adoption. *J. Hum. Cap.* **3**, 289–322 (2009).

[19] Cojuharenco, I. & Karelaia, N. When leaders ask questions: Can humility premiums buffer the effects of competence penalties?. *Organ. Behav. Hum. Decis. Process.* **156**, 113–134 (2020).

[20] Kämmer, J. E., Choshen-Hillel, S., Müller-Trede, J., Black, S. L. & Weibler, J. A systematic review of empirical studies on advice-based decisions in behavioral and organizational research. *Decision* **10**, 107–137 (2023).

[21] Weiner, B. An attributional theory of achievement motivation and emotion. *Psychol. Rev.* **92**, 548–573 (1985). 3903815

[22] Dai, T. & Singh, S. Artificial intelligence on call: the physician’s decision of whether to use AI in clinical practice. *J. Mark. Res.* (forthcoming) 10.1177/00222437251332898 (2025).

[23] Topol, E. J. High-performance medicine: the convergence of human and artificial intelligence. *Nat. Med* **25**, 44–56 (2019). 30617339 10.1038/s41591-018-0300-7

[24] Dai, T. & Singh, S. Conspicuous by its absence: diagnostic expert testing under uncertainty. *Mark. Sci.* **39**, 540–563 (2020).

[25] Rosenbaum, L. The less-is-more crusade — are we overmedicalizing or oversimplifying?. *N. Engl. J. Med* **377**, 2392–2397 (2017). 29236644 10.1056/NEJMms1713248

[26] Nickerson, R. S. Confirmation bias: a ubiquitous phenomenon in many guises. *Rev. Gen. Psychol.* **2**, 175–220 (1998).

[27] Tversky, A. & Kahneman, D. Judgment under Uncertainty: Heuristics and Biases: Biases in judgments reveal some heuristics of thinking under uncertainty. *Science* **185**, 1124–1131 (1974). 17835457 10.1126/science.185.4157.1124

[28] Norman, G. Likert scales, levels of measurement and the “laws” of statistics. *Adv. Health Sci. Educ.* **15**, 625–632 (2010). 10.1007/s10459-010-9222-y 20146096

[29] Herrmann, A. et al. Acceptability of health-only versus climate-and-health framings in lifestyle-related climate-sensitive health counselling: results of a randomised survey experiment in Germany. *Lancet Planet. Health* **9**, e456–e466 (2025). 40516537 10.1016/S2542-5196(25)00110-X

[30] Sagona, M., Dai, T., Macis, M. & Darden, M. Trust in AI-assisted health systems and AI’s trust in humans. *npj Health Syst.* **2**, 10 (2025). 10.1038/s44401-025-00016-5 PMC13354213 42527442

[31] Daugherty, S. R., Baldwin, D. C. Jr & Rowley, B. D. Learning, satisfaction, and mistreatment during medical internship: a national survey of working conditions. *JAMA* **279**, 1194 (1998). 9555759 10.1001/jama.279.15.1194

[32] Gettel, C. J. et al. Calculation of overall hospital quality star ratings with and without inclusion of the peer grouping step. *JAMA Netw. Open* **7**, e2411933 (2024). 38753326 10.1001/jamanetworkopen.2024.11933 PMC11099678

[33] Martinez, K. A. et al. The association between physician race/ethnicity and patient satisfaction: an exploration in direct to consumer telemedicine. *J. Gen. Intern. Med.* **35**, 2600–2606 (2020). 32632788 10.1007/s11606-020-06005-8 PMC7459065

[34] Siddiqui, Z. K. et al. Comparison of services available in 5-star and non–5-star patient experience hospitals. *JAMA Intern Med* **179**, 1429 (2019). 31180447 10.1001/jamainternmed.2019.1285 PMC6563548

[35] Stokes, D. C. et al. Association between crowdsourced health care facility ratings and mortality in US counties. *JAMA Netw. Open* **4**, e2127799 (2021). 34665240 10.1001/jamanetworkopen.2021.27799 PMC8527362

[36] Hayes, A. F. *Introduction to Mediation, Moderation, and Conditional Process Analysis: A Regression-Based Approach*. (Guilford Press, New York, 2018).

