# Supplementary material for: Peer perceptions of clinicians using generative AI in medical decision-making
Source: NPJ Digit Med. 2025 Aug 18;8:530. doi: 10.1038/s41746-025-01901-x (PMC12361413; doi:10.1038/s41746-025-01901-x)
Supplement: Supplementary file 1 — Supplementary Information [file 41746_2025_1901_MOESM1_ESM.docx]

**Supplementary Note 1 | Vignette Scenarios**

**Control Condition**

Please read the following scenario and respond to a few questions below:

*Dr. Miller is seeing an adult patient with type 2 diabetes. After speaking with the patient, Dr. Miller reviews blood sugar data, weight, blood pressure, and recent lab results. The blood pressure has increased and the patient has gained weight.*

*Dr. Miller suggests that she’d like to consider changing the diabetes medication regimen and introduce a medication that might help with weight loss and have long-term cardioprotective benefits. Dr. Miller recommends starting a new medication, called an SGLT2-inhibitor, as it may have a favorable impact on weight, blood pressure, and heart disease. Dr. Miller explains the risks and benefits of this medication and provides a prescription. She also provides lifestyle counseling on nutrition, activity, and weight loss to complement the medication changes.*

**GenAI-primary Condition**

Please read the following scenario and respond to a few questions below:

*Dr. Miller is seeing an adult patient with type 2 diabetes. After speaking with the patient, Dr. Miller reviews blood sugar data, weight, blood pressure, and recent lab results. The blood pressure has increased and the patient has gained weight.*

*Dr. Miller enters the clinical data and test results into ChatGPT (without patient identifying information), a widely popular artificial intelligence program, and queries to get a recommendation on which new medication would be best for this patient’s diabetes management. ChatGPT provides Dr. Miller with the following answer, “I'm not a doctor, but based on the information provided, I would consider introducing an SGLT2-inhibitor as it may have a favorable impact on weight, blood pressure, and heart disease.” Dr. Miller recommends starting an SGLT2-inhibitor as it may have a favorable impact on weight, blood pressure, and heart disease. Dr. Miller explains the risks and benefits of this medication and provides a prescription. She also provides lifestyle counseling on nutrition, activity, and weight loss to complement the medication changes.*

**GenAI-verify Condition**

Please read the following scenario and respond to a few questions:

*Dr. Miller is seeing an adult patient with type 2 diabetes. After speaking with the patient, Dr. Miller reviews blood sugar data, weight, blood pressure, and recent lab results. The blood pressure has increased and the patient has gained weight.*

*Dr. Miller suggests that she’d like to consider changing the diabetes medication regimen and introduce a new medication that might help with weight loss and have long-term cardioprotective benefits. Dr Miller wants to start an SGLT2-inhibitor because it has favorable cardiovascular benefits and can result in weight loss. She queries ChatGPT to double-check her choice of medication to start an SGLT-2 inhibitor. ChatGPT returned the following answer: “I'm not a doctor, but an SGLT-2 inhibitor would be a reasonable addition for this patient with type 2 diabetes and high blood pressure and increased weight, as long as kidney function is adequate.” Dr. Miller explains that she uses ChatGPT as an additional level of verification, and the result is consistent with her recommendation. Dr. Miller explains the risks and benefits of this medication and provides a prescription. She also provides lifestyle counseling on nutrition, activity, and weight loss to complement the medication changes.*
